# Addition of Berberine to 5-Aminosalicylic Acid for Treatment of Dextran Sulfate Sodium-Induced Chronic Colitis in C57BL/6 Mice

**DOI:** 10.1371/journal.pone.0144101

**Published:** 2015-12-07

**Authors:** Yan-hong Li, Man Zhang, Hai-tao Xiao, Hai-bo Fu, Alan Ho, Cheng-yuan Lin, Yu Huang, Ge Lin, Zhao-xiang Bian

**Affiliations:** 1 Lab of Brain and Gut Research, Hong Kong Chinese Medicine Research Center, School of Chinese Medicine, Hong Kong Baptist University, Hong Kong, China; 2 School of Biomedical Sciences, Chinese University of Hong Kong, Hong Kong, China; National Cancer Institute, UNITED STATES

## Abstract

Ulcerative colitis (UC) is a common chronic remitting disease but without satisfactory treatment. Alternative medicine berberine has received massive attention for its potential in UC treatment. Conventional therapies with the addition of berberine are becoming attractive as novel therapies in UC. In the present study, we investigated the preclinical activity of a conventional oral 5-aminosalicylic acid (5-ASA) therapy plus berberine in experimental colitis. A subclinical dose of 5-ASA (200 mg/kg/day) alone or 5-ASA plus berberine (20 mg/kg/day) was orally administered for 30 days to C57BL/6 mice with colitis induced by three cycles of 2% dextran sulfate sodium (DSS). The disease severity, inflammatory responses, drug accumulation and potential toxicity of colitis mice were examined. The results showed that comparing to 5-ASA alone, 5-ASA plus berberine more potently ameliorated DSS-induced disease severity, colon shortening, and colon histological injury. Further, the up-regulation in mRNA level of colonic TNF-α as well as NFκB and JAK2 phosphorylation caused by DSS were more pronouncedly reversed in animals treated with the combination therapy than those treated with 5-ASA alone. Moreover, the addition of berberine to 5-ASA more significantly inhibited lymphocyte TNF-α secretion of DSS mice than 5-ASA alone. In the meanwhile, no extra drug accumulation or potential toxicity to major organs of colitis mice was observed with this combination treatment. In summary, our studies provide preclinical rationale for the addition of berberine to 5-ASA as a promising therapeutic strategy in clinic by reducing dose of standard therapy.

## Introduction

Ulcerative colitis (UC) is a chronic relapsing and remitting inflammatory disease of the colon which causes severe diarrhea, abdominal pain and increases the risk of colorectal cancer [1]. UC mostly affects the young and middle-aged and up to 18% of patients suffer chronic active disease associated with significant morbidity and loss of productivity, which generally requires lifelong treatment [1]. With increasing incidence, UC has a significant impact on patient’s lives and brings enormous cost burden on healthcare system. However, up to date, there is no cure for UC; the overall goals are to induce and maintain remission, and to prevent complications [1]. It is thus urgent to establish new treatment strategies for reducing UC.

In current practice, 5-aminosalicylic acid (5-ASA) still represents the cornerstone of first-line therapy for mild-to-moderate UC even after several decades of application [2,3]. 5-ASA administration *in vivo* can reduce the inflammatory responses through two major mechanisms: inhibition of prostaglandin E2 (PGE2) production via cyclooxygenase-2 (COX-2) and inhibition of nuclear factor κB (NFκB) activation [4,5]. 5-ASA and its derivatives have been widely studied in inducing and maintaining remission in UC [2,3]. Unfortunately, the general response rate is only 70%−80% and the relapse rate largely varies [6]. Therefore, 5-ASA agents often require multiple doses daily and many pills, which usually results in a low patient compliance in the long-term [6]. It is necessary to search for strategies to improve the therapeutic effects of 5-ASA.

Immunomodulatory agents originated from alternative medicine, particularly Chinese herbal medicines (CHM), represent a promising class of novel agents for UC therapy [7]. Berberine is a major bioactive component of *Coptidis Rhizome* which is commonly used in the CHM formulas for UC patients [8]. It possesses multiple pharmacological effects such as antidiarrheic and anti-inflammatory activities and has been proved to be safe along a chronic administration [9,10]. Recently, berberine was demonstrated to be able to inhibit cytokine responses via activator of transcription (STAT)-3 [11]. STAT3 is key molecular in the janus kinase (JAK)-STAT (JAK/STAT) signaling pathway which is central in mediating cytokine responses under chronic conditions of UC [12]. Interestingly, many recent studies have demonstrated that berberine has excellent protective effects against experimental models of UC [13–16]. Moreover, a clinical trial has been just initiated to evaluate the efficacy and safety of berberine in UC patients [17]. Based on above knowledge, it is rational to hypothesize that with anti-UC activity combined with pharmacological safety, berberine might allow a reduction in the amount of 5-ASA required for treatment and so increase the adherence of patient upon a chronic administration.

Therefore, our present study aimed to evaluate whether the addition of berberine to a subclinical dose of 5-ASA showed effect in mice with DSS-induced chronic relapsing colitis. In addition to characterizing its molecular mechanism of their anti-UC activity, we defined the possible drug accumulation and potential toxicity associated with this dual therapy. Our data may inform the design of a future clinical trial evaluating 5-ASA plus berberine in UC patients.

## Materials and Methods

### Chemicals and reagents

**Fig 1:**
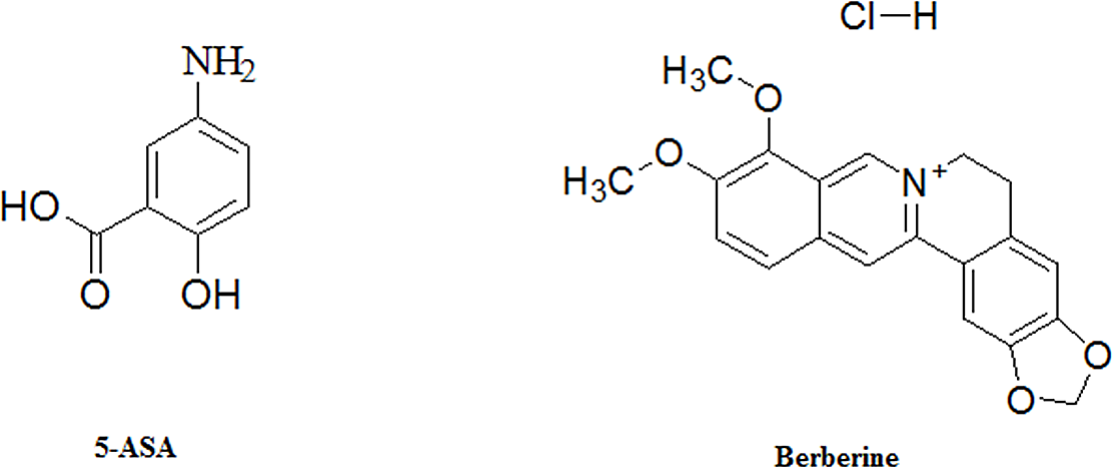
Chemical structure of 5-ASA and berberine.

5-ASA, N-acetyl-5-aminosalicylic acid (Ac-5-ASA), lithium chloride, phorbol myristate acetate (PMA) and ionomycin were purchased from Sigma Chemical Co. (St. Louis, MO, USA). Berberine hydrochloride was purchased from Shenzhen ChemStrong Scientific Co., Ltd (Shenzhen, China) at the highest available purity (≥ 95%). The structure of 5-ASA and berberine hydrochloride is shown in Fig 1. Dextran sulfate sodium (DSS) was the product of MP Biomedicals (MW; 36,000−50,000, MP Biomedicals, Solon, OH, USA). HPLC grade of acetonitrile, methanol and formic acid were purchased from Merck (Darmstadt, Germany). Deionized water was purified by the Millipore water purification system (Millipore, Milford, MA, USA). All other reagents used were of analytical grade.

### Experimental animals

Male, 12-week old C57BL/6 mice weighing 23 to 25 g obtained from The Chinese University of Hong Kong were used in this study. The animals were housed in rooms at a controlled temperature and with light/dark (12 hr/12 hr) cycles. They were fed standard mice chow pellets and had access to filtered water supplied in bottles. All animal care and experimental procedures were approved by the Animal Care and Use Committee at Hong Kong Baptist University.

### Induction of colitis and treatment

In order to evaluate the damage progression overtime, experimental chronic colitis was induced by giving mice three cycles of DSS with protocol modified according to the description of Wirtz *et al* [18]. Briefly, DSS was dissolved in drinking water at a concentration of 2.0% (w/v). Each cycle consisted of 2% DSS for five days followed by drinking water for 14 days. The first day and the last day of treatment were designated as day 0 and day 43, respectively. All mice after receiving one cycle of DSS developed colitis and they were randomly divided into three groups with eight mice in each group: DSS, 5-ASA (200 mg/kg) alone and 5-ASA (200 mg/kg) plus berberine (20 mg/kg) (*n* = 8 for each group). The animals within each group showed a comparable severity of colitis and the amount of consumed drinking water was similar in all experimental groups. 5-ASA or 5-ASA plus berberine was freshly prepared and administered orally once daily in a volume of 200 μl in parallel with or without DSS-feeding for a total of 30 days. A group of mice designated as water group received filtered water alone throughout the experiment course (*n* = 8).

### Determination of clinical score, colon length and colon histological changes

Body weight, stool consistency and occult blood or the presence of gross blood per rectum were determined according to the described criterion [18]. Briefly, weight loss of 1–5%, 5–10%, 10–20%, and >20% was scored as 1, 2, 3, and 4, respectively. For stool consistency, 0 was scored for well-formed pellets, 1 for soft but formed stools, 2 for pasty and semi-formed stools, which did not stick to the anus, and 3 for liquid stools that remained adhesive to the anus. Bleeding was scored 0 for no blood in hemoccult, 1 for positive hemoccult, 2 for gross bleeding in facet and 3 for gross bleeding from the rectum. Weight loss, stool consistency, and stool bleeding sub-scores were added, resulting in a total clinical score ranging from 0 (healthy) to 10 (maximal activity of colitis).

By the end of treatment, the animals were fasted for 24 hours and then anaesthetized by intraperitoneal injection of chloral hydrate. Thereafter, blood was collected from eye before animals were sacrificed by cervical dissociation. Post mortem the entire colon was removed from the caecum to the anus and the colon length was measured. Afterwards, the colon of each animal was divided according to their length: the distal 10% was fixed in 10% buffered formalin for histological examination, the next 40% snap-frozen in liquid nitrogen for cytokine mRNA quantification, another 40% was dissected out for Western bolt assay, and the proximal 10% was used for LC-MS analysis. Rings of the transverse part of the colon fixed in formalin were embedded in paraffin and sections at 5 μm thickness were stained with hematoxylin & eosin (H&E).

### Colon mRNA extraction and quantitative real-time reverse-transcription polymerase chain reaction (RT-qPCR)

Total RNA was isolated using Trizol (Invitrogen, La Jolla, CA, USA) and subjected to mRNA purification using lithium chloride precipitation as recommended by Viennois *et al* [19]. Reverse transcription was performed with the cDNA Synthesis Kit (Takara Biotech, Japan). The mRNA expression of mucin genes, cytokines and the internal control glyceraldehyde 3-phosphate dehydrogenase (Gapdh) was measured by RT-qPCR performed with the 7500 Real Time PCR System (Applied Biosystems, CA, USA). Mouse mRNA primers used in analysis were purchased from Life Technologies (Waltham, MA, USA) and the sequences are listed as follows: MUC1: 5'-TCT CCA GCC ACC AGC CCT CTA A-3', 5'-TGG CCA TGG TAG GAG AAA CAG G-3'; MUC2: 5'-GGG AGG GTG GAA GTG GCA TTG T-3', 5'-TGC TGG GGT TTT TGT GAA TCT C-3'; MUC4: 5'-CAT ATT CAA TAC CAC CGG TGT TC-3', 5'-AAG GAT GGA ATT GGT GCT TTG TC-3'; COX-2: 5'-TTC TCT ACA ACA ACT CCA TCC TC-3', 5'-GCA GCC ATT TCC TTC TCT CC-3'; TNF-α:5'-GAC CCT CAC ACT CAG ATC ATC CTT CT-3', 5'-ACG CTG GCT CAG CCA CTC-3'; IL-12b: 5'-TAA CCA GAA AGG TGC GTT CC-3', 5'-CTT TCC AAC GTT GCA TCC TA-3'; Gapdh: 5'-ATG TTC CAG TAT GAC TCC ACT CAC G-3', 5'-AAG ACA CCA GTA GAC TCC ACG ACA-3'. The mRNA expression level in the water group was set as 100%, and mRNA expression levels in treated mice were compared with that of the water group.

### Western blot

Frozen colon samples were homogenized in PBS with 1% phosphatase inhibitor cocktail (Sigma-Aldrich St. Louis, MO, USA). Homogenates were centrifuged (12,000×g, 15 min, 4°C) and the supernatants were collected. Protein concentration was determined following Bradford's colorimetric method. Aliquots of supernatants containing equal amounts of protein (20 μg) were separated on 10% SDS-PAGE gel and then transferred to a PDF membrane (Hybond, GE Healthcare, UK). After blocking, membranes were incubated with specific primary antibodies (NFκB, IKKα, IKKβ, JAK2, STAT1, STAT3 or β-actin and phosphorylated NFκB (p-NFκB), IKKαβ (p-IKKαβ), JAK2 (p-JAK2), STAT1 (p-STAT1), STAT3 (p-STAT3)) (Cell Signaling Technology, MA, USA) at the dilution of 1:2000. After three washes, membrane was then incubated with the secondary horseradish peroxidase linked anti-rabbit or anti-mouse IgG (Santa Cruz Biotechnology, Inc, CA, USA). Immunodetection was performed using chemiluminescence light-detecting kit (West Pico Chemiluminescent Substrate, Pierce, Rockford, IL, USA). Densitometry data were measured following normalization to the control by Adobe® Photoshop CS3 and Image J software. The relative density of the signaling band on Western blot was compared with the β-actin band in each group. The ratio in the water group was set as 1, and ratios in treated mice were compared with that of the water group.

### Lymphocyte culture

Spleens were aseptically removed from water or DSS-treated mice at the end of study. Lymphocyte suspensions were prepared according to standard procedures using lympholyte-M (Cedarlane Labs, Hornby, Ontario, Canada). Cells were washed twice in RPMI-1640 (Gibco, Carlsbad, CA USA), resuspended in medium containing 10% fetal bovine serum (FBS) (Gibco, Carlsbad, CA USA), and cultured at concentration of 1.0 ×10^6^ cells/ml in 24-well plates. Cultures were incubated at 37°C in a humidified atmosphere with 5% CO_2_ for 20 hr in the presence of 1 μM 5-ASA, 5-ASA plus 1 μM berberine or absence of any drug, PMA (50 ng/ml) and ionomycin (500 ng/ml) were then added for additional 4 hr. At the end of the incubation period the culture medium were frozen at –70°C until cytokine measurement.

### Enzyme-linked immunosorbent assay (ELISA)

Concentrations of PGE2 and TNF-α were measured using commercial mouse ELISA kits according to the manufacturer's instructions (eBioscience, San Diego, CA, USA).

### Determination of 5-ASA and its metabolite Ac-5-ASA by LC-MS/MS

Serum samples (50 μl) were added to 400 μl of methyl alcohol for deproteinization. After centrifugation at 12,000g for 10 min, the upper organic layer was transferred into a polythene tube and dried using centrifugal vacuum at 37°C. The dried residue was dissolved in 200 μl of the mobile phase. The colon slices (50 mg) were mixed with 300 μl of normal saline after weighing and then homogenized (IKA-T10 homogenizer; IKA, Staufen, Germany) in an ice-bath environment. The other preparations were handled the same as the serum samples. Two microliters of each sample were injected for LC-MS/MS analysis (API 3200; Applied Biosystems, Foster City, CA, USA) using ultra-high-performance liquid chromatography (UHPLC-QQQ-MS) system (Agilent, Santa Clara, CA, USA) and methods modified according to a previous report [20].

### Histological evaluation of potential toxicity

Mice were sacrificed following blood collection. The positions, shapes, sizes and colors of internal organs were recorded. The spleen, lung, liver, and brain were collected and preserved in 10% neutral formalin. The tissues were then embedded in paraffin, sectioned, and stained with H&E.

### Data analysis

All data are presented as the means ± standard deviation (SD) of at least three biological replicates. The clinical score data were tested for statistically significant differences in the mean values by two-way ANOVA. To detect the differences in the presence of a significant two-way ANOVA, a Tukey post hoc analysis was performed. Remaining data between multiple groups were compared using one-way ANOVA followed by Duncan post-hoc test. Statistical analyses were performed using Prism 6 for Windows software (GraphPad Software, Inc., San Diego, CA, USA). *P*-values of 0.05 or less were considered statistically significant.

## Results

### Clinical score and colon length

**Fig 2:**
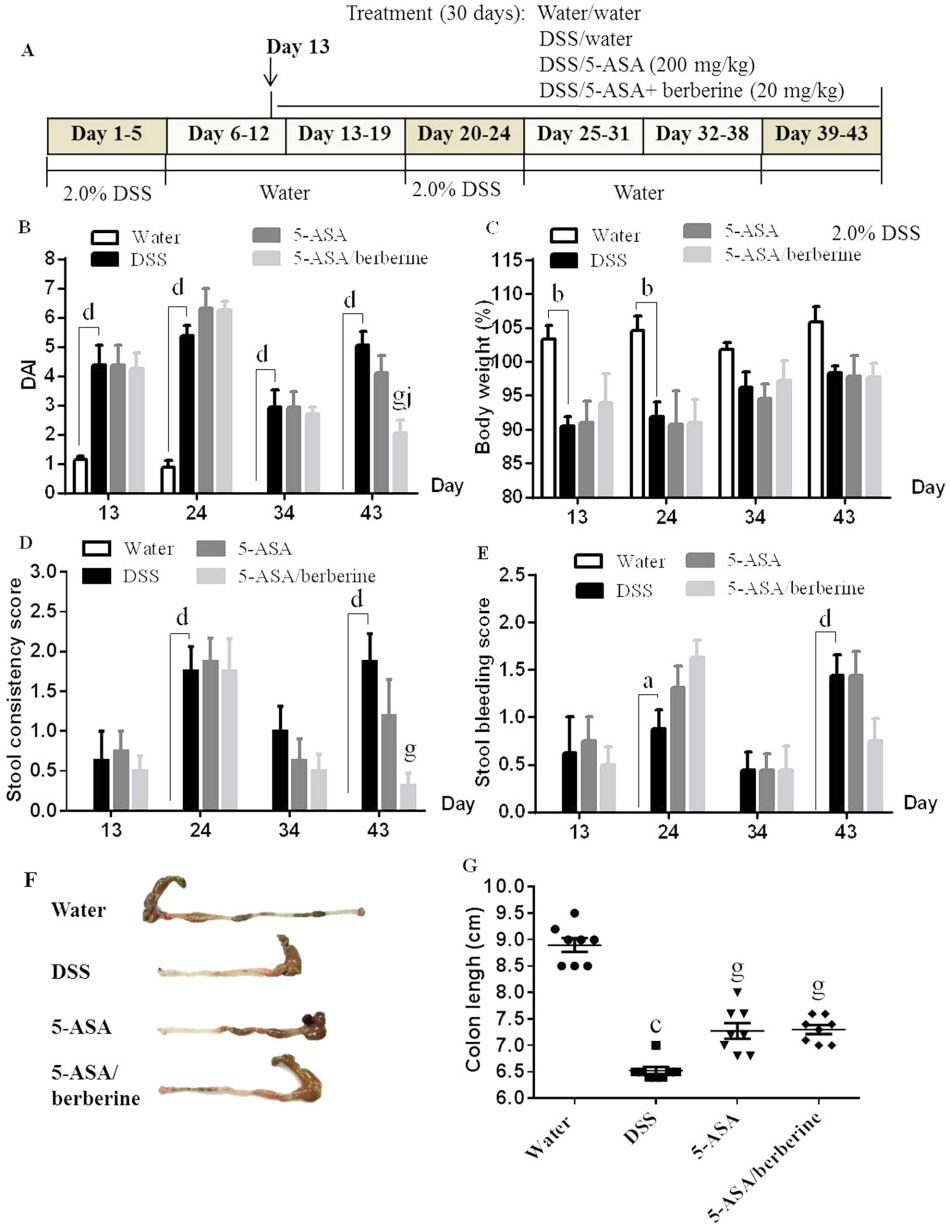
Effects of the addition of berberine to 5-ASA on clinical score and colon length of DSS-induced chronic relapsing colitis mice. Experimental colitis was induced by giving mice three cycles of DSS. Each cycle consisted of 2% DSS for five days followed by drinking water for 14 days. The first and last day of DSS treatment were designated as days 0 and 43, respectively. From day 13, experimental mice were treated daily with 5-ASA (200 mg/kg) alone or 5-ASA plus berberine hydrochloride (20 mg/kg) through gavage of 0.2 ml of the respective solution during the 30 days of concomitant DSS-colitis induction. Control mice received water alone. The experimental protocol for DSS-induced colitis in mice and the administration of berberine is shown in (A). The degree of colitis was quantified by the disease activity index (DAI) (B) by assessing loss of body weight (C), scores of stool consistency (D) and stool bleeding (E). The colon was collected and its length was measured after mice were sacrificed by the end of treatment (F and G). ^a^ *P* < 0.05, ^b^ *P* < 0.01, ^c^ *P* < 0.001, ^d^ *P* < 0.0001 versus water; ^g^ *P* < 0.001 versus DSS; ^j^ *P* < 0.01 versus 5-ASA alone (n = 8 mice per treatment group).

The experimental protocol is shown in Fig 2A. Mice exposed to 2.0% DSS developed signs of colitis as expressed by comparable clinical score of greater than 3 from day 5 onward before drug treatment between groups (full data not shown). Significant increases in the DAI at all four tested time points (Fig 2B), in loss of body weight at two early time points (day 13 and 24) (Fig 2C), as well as in scores of stool consistency and stool bleeding at two DSS-treated time points (day 24 and 43) (Fig 2D and 2E) were observed in animals treated with DSS when compared to water control mice. Administration of 200 mg/kg/d 5-ASA alone clearly decreased the DAI and stool consistency score only at the end of treatment (Day 43) when compared to the untreated DSS animals, but the difference did not reach significance (*n* = 8; Fig 2B and 2D). Treatment with 200 mg/kg 5-ASA plus 20 mg/kg berberine was also only effective after 30-day administration, while it resulted in a much lower DAI, and scores of both stool consistency and stool bleeding (*n* = 8; Fig 2B, 2D and 2E). Further, this difference in DAI between 5-ASA alone and 5-ASA plus berberine treatment groups was significant (Fig 2B). The water control animals, which received regular drinking water did not develop signs of colitis during the whole experimental course (*n* = 8) (all clinical scores < 1.5 from days 1 to 43, detailed data not shown).

Colon length is an indirect and reproducible surrogate for colitis severity of colonic inflammation [21]. Three cycles of DSS challenge induced a significant reduction in colon length (colon shorting) when compared to water control animals (*n* = 8; Fig 2F and 2G). The administration of 5-ASA alone partially but significantly reversed the DSS-induced colon shorting (*n* = 8; Fig 2F and 2G). DSS-exposed animals treated with 5-ASA plus berberine also showed significant effect on reducing colon shorting comparing with animals treated with DSS (*n* = 8; Fig 2F and 2G). When compared to 5-ASA alone, the dual therapy did not significantly improved the colon length, but resulted in a largely minimized variation of colon length between individual animals (Fig 2G).

### Colon histological changes and mucin genes expression

**Fig 3:**
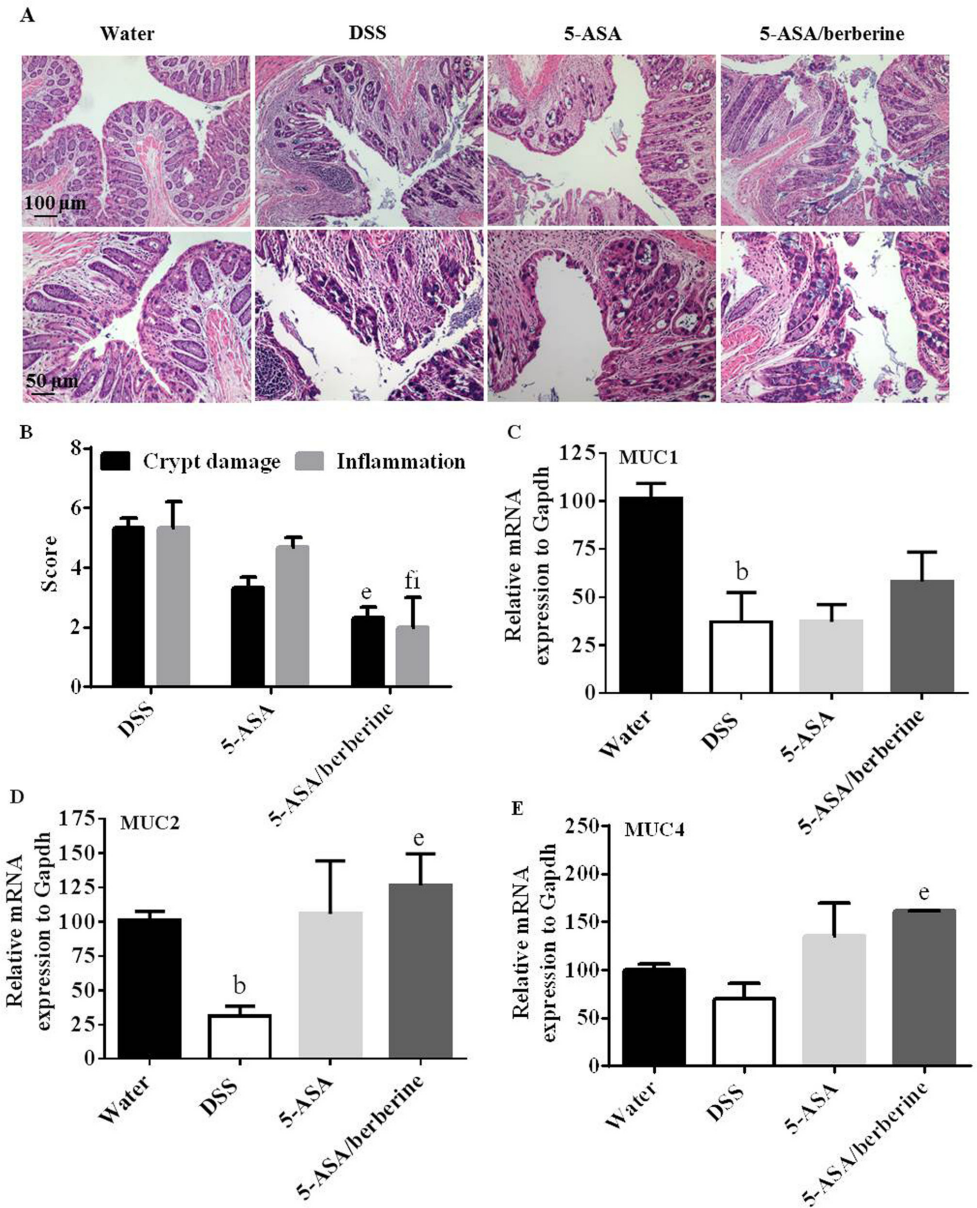
Effects of the addition of berberine to 5-ASA on colon tissue injury and mRNA expressions of mucin genes of DSS-induced chronic relapsing colitis mice. Experimental colitis was induced by giving mice three cycles of DSS. Each cycle consisted of 2% DSS for five days followed by drinking water for 14 days. The first and last day of DSS treatment were designated as days 0 and 43, respectively. From day 13, experimental mice were treated daily with 5-ASA (200 mg/kg) alone or 5-ASA plus berberine hydrochloride (20 mg/kg) through gavage of 0.2 ml of the respective solution during the 30 days of concomitant DSS-colitis induction. Control mice received water alone. At the end of treatment, mice were killed and the colon sections were collected and subjected to paraffin embedding and later hematoxylin and eosin (H&E) staining for light microscopic assessment (A) and colon injury/inflammation scoring (B). Scale bar = 50 or 100 μm. The colonic mRNA expression of MUC1 (C), MUC2 (D), MUC4 (E) and the housekeeping gene Gapdh in colitis mice was determined by RT-qPCR. The cytokine mRNA expression level in the water group was set as 100%, and mRNA expression levels in treated mice were compared with the water group. ^b^ *P* < 0.01 versus water; ^e^ *P* < 0.05, ^f^ *P* < 0.01 versus DSS; ^h^ *P* < 0.05 versus 5-ASA alone (n = 4 mice per treatment group).

Histology of rings of the transverse part of the colon in DSS-exposed mice revealed multiple erosive lesions including losses of crypt and columnar epithelium and inflammatory cell infiltration (*n* = 4; Fig 3A). Treatment with 5-ASA showed a tendency to decrease the colon tissue injury as compared to the DSS control (*n* = 4; Fig 3A and 3B). Animals treated with 5-ASA plus berberine had a significantly improved histology of colon and the inflammation score was more significantly decreased when compared to 5-ASA alone (*n* = 4; Fig 3A and 3B). In the water control group not exposed to DSS, no histologic signs of inflammation were detected (*n* = 4; Fig 3A).

On the mucosal surface, mucins constitute a layer of mucus lining the gastrointestinal tract to form a lubricant and physical barrier. The changes in mucin quantity have been proved involved in ulcerative injury during UC pathogenesis [22–24]. The mRNA expression of colon mucin genes revealed that MUC1, MUC2 and MUC4 were all reduced by DSS challenge (*n* = 4; Fig 3C–3E). These reductions were slightly alleviated by 5-ASA alone, while significantly alleviated by 5-ASA plus berberine when comparing to DSS control (*n* = 4; Fig 3C–3E). Unexpectedly, the dual therapy did not significantly alleviate the reductions of mucin genes expressions when compared to 5-ASA alone (*n* = 4; Fig 3C–3E).

### Pro-inflammatory responses in colon of DSS-induced colitis mice

**Fig 4:**
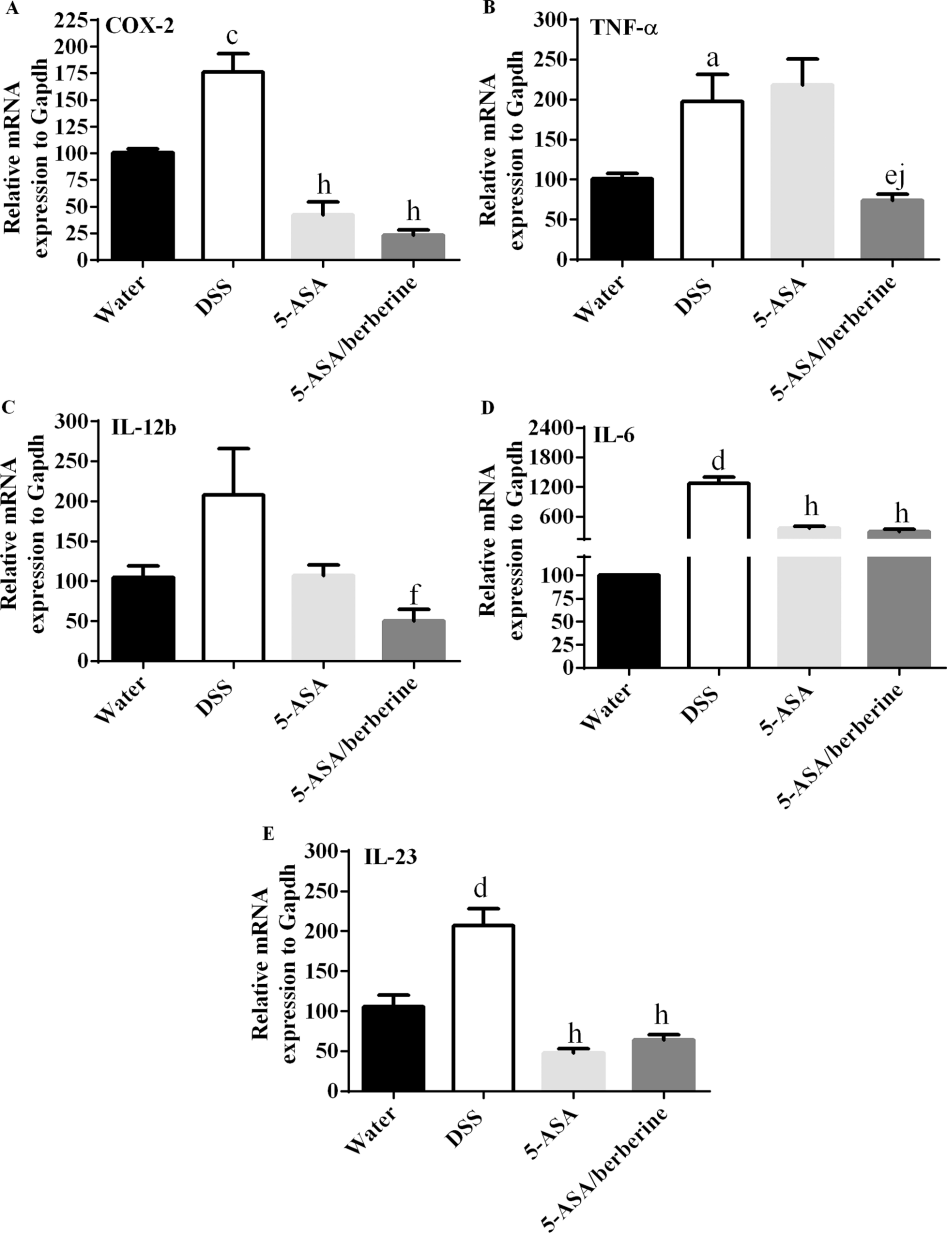
Effects of the addition of berberine to 5-ASA on pro-inflammatory responses of colon in DSS-induced chronic relapsing colitis mice. Experimental colitis was induced by giving mice three cycles of DSS. Each cycle consisted of 2% DSS for five days followed by drinking water for 14 days. The first and last day of DSS treatment were designated as days 0 and 43, respectively. From day 13, experimental mice were treated daily with 5-ASA (200 mg/kg) alone or 5-ASA plus berberine hydrochloride (20 mg/kg) through gavage of 0.2 ml of the respective solution during the 30 days of concomitant DSS-colitis induction. Control mice received water alone. At the end of treatment, mice were killed and the colon mRNA was isolated for real-time PCR analysis of the mRNA abundance of COX-2 (A), TNF-α (B), IL-12b (C), IL-6 (D), IL-23 (E) and the housekeeping gene Gapdh. The cytokine mRNA expression level in water group was set as 100%. mRNA expression levels in DSS, 5-ASA or 5-ASA plus berberine-treated groups were compared with the water group. ^a^ *P* < 0.05, ^c^ *P* < 0.001, ^d^ *P* < 0.0001 versus water; ^e^ *P* < 0.05, ^f^ *P* < 0.01, ^h^ *P* < 0.0001 versus DSS; ^j^ *P* < 0.01 versus 5-ASA alone (*n* = 4 mice per treatment group).

To explore how above beneficial effects generated the mRNA levels of the pro-inflammatory cytokines in the colonic tissue of animals treated with 5-ASA alone or 5-ASA plus berberine after DSS administration were measured. The results showed that DSS-induced colitis was accompanied by a significant up-regulation of all tested cytokines mRNA in the colons of animals (n = 4; Fig 4A–4E). Animals treated with 5-ASA alone showed a distinguished decrease in mRNA levels of COX-2, IL-6 and IL-23, but no significant decrease in TNF-α or IL-12b when compared to the DSS control (Fig 4A–4E). Interestingly, a dramatic inhibition of the five cytokines mRNA were observed after 5-ASA plus berberine treatment when compared to DSS control (*n* = 4; Fig 4A–4E). Especially, the dual therapy significantly inhibited the increase of TNF-α mRNA when compared with 5-ASA alone (n = 4; Fig 4B).

### NFκB and JAK/STAT pathways activation in colon of DSS-induced colitis mice

**Fig 5:**
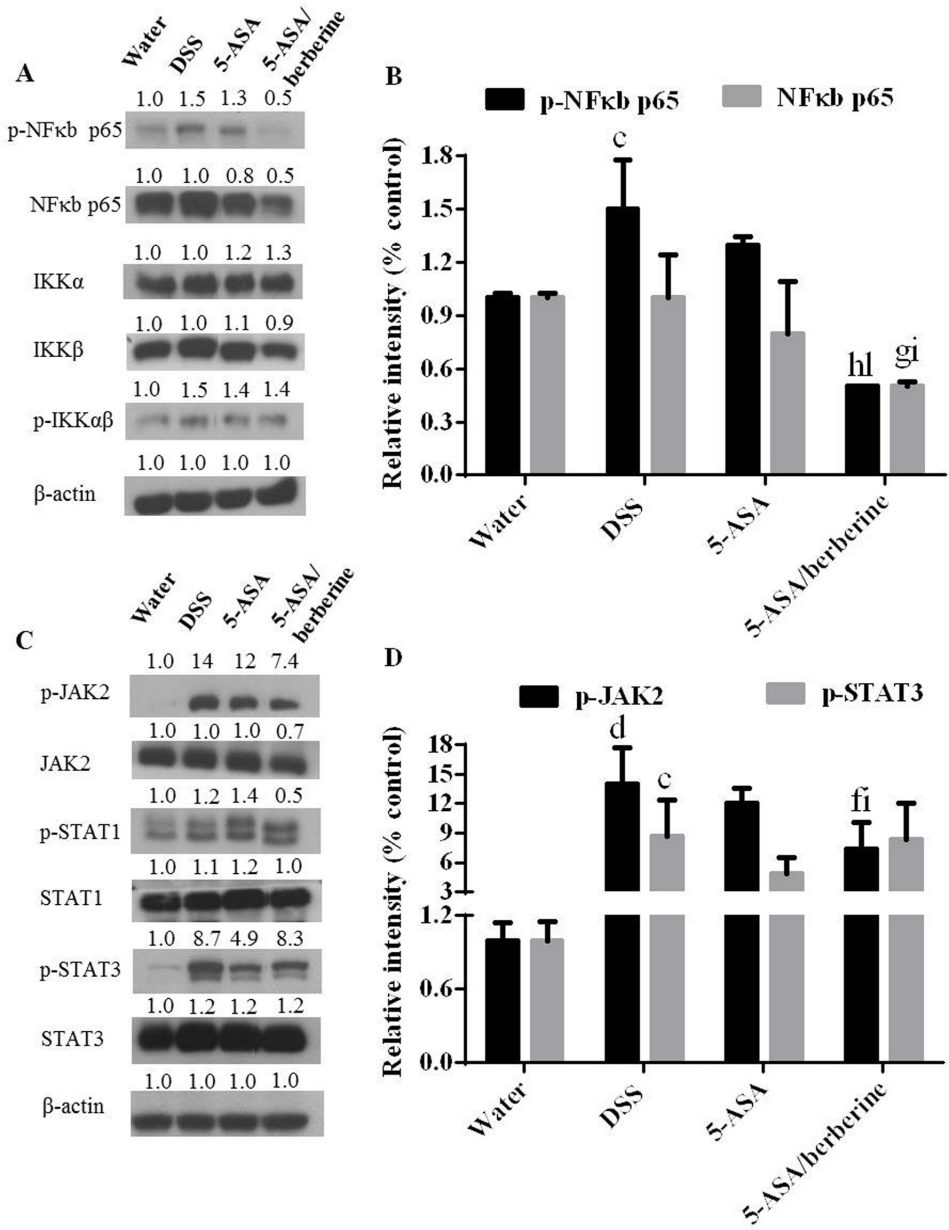
Effects of the addition of berberine to 5-ASA on activated NFκB and JAK/STAT pathways of colon in DSS-induced chronic relapsing colitis mice. Experimental colitis was induced by giving mice three cycles of DSS. Each cycle consisted of 2% DSS for five days followed by drinking water for 14 days. The first and last day of DSS treatment were designated as days 0 and 43, respectively. From day 13, experimental mice were treated daily with 5-ASA (200 mg/kg) alone or 5-ASA plus berberine hydrochloride (20 mg/kg) through gavage of 0.2 ml of the respective solution during the 30 days of concomitant DSS-colitis induction. Control mice received water alone. At the end of treatment, mice were killed and the colon total cellular lysates were prepared for detecting the amounts of the indicated signals of NFκB (A and B) and JAK/STAT (C and D) pathways by Western blot analysis. Blotting for β-actin was used as a protein loading control. The relative density of the target protein bands on Western blot was compared with the β-actin band in each group. The density ratio in the water group was set as 1, and ratios of other three groups were compared with that of the water group. ^c^ *P* < 0.001 and ^d^ *P* < 0.0001 versus water; ^f^ *P* < 0.01, ^g^ *P* < 0.001, ^h^ *P* < 0.0001 versus DSS; ^i^ *P* < 0.05 and ^l^ *P* < 0.0001 versus 5-ASA alone (*n* = 4 mice per treatment group).

Our findings described above support a more potently negative regulatory role of 5-ASA plus berberine in pro-inflammatory responses. To define the underlying mechanism, two key signaling pathways NFκB and JAK/STAT, which play roles in UC pathogenesis and reported involved in either 5-ASA or berberine bioactivity were investigated [11,12,25]. Interestingly, we found that the combo treatment more significantly downregulated both the NFκB p65 and phosphorylation status of it with other signals of this pathway and β-actin not significantly affected (*n* = 4; Fig 5A and 5B). In addition, in consistence with reductions in the above-described cytokines, DSS induction-induced elevated phosphorylation of JAK2 was significantly decreased upon treatment with 5-ASA plus berberine when compared to DSS or 5-ASA alone treatment group (Fig 5C and 5D). Collectively, these results indicate that the additional efficacy of combined treatment was attributable, at least in part, to inhibition of the NFκB and JAK/STAT signaling.

### PGE2 production and TNF-α secretion by re-stimulated lymphocyte of DSS-induced colitis mice

5-ASA and berberine have been proved to exert anti-inflammatory property via inhibiting PGE2 and/or TNF-α. Thereby we evaluated the direct effects of 5-ASA alone or its combination with berberine on PGE2 production and TNF-α secretion ex-vivo. Lymphocytes isolated from the spleens of water or DSS-treated mice were isolated and cultured for 20 hr with vehicle control, 5-ASA alone or 5-ASA plus berberine treatment. Cells were then re-stimulated by PMA (25 ng/ml) plus ionomycin (500 ng/ml) for additional 4 hr.

**Fig 6:**
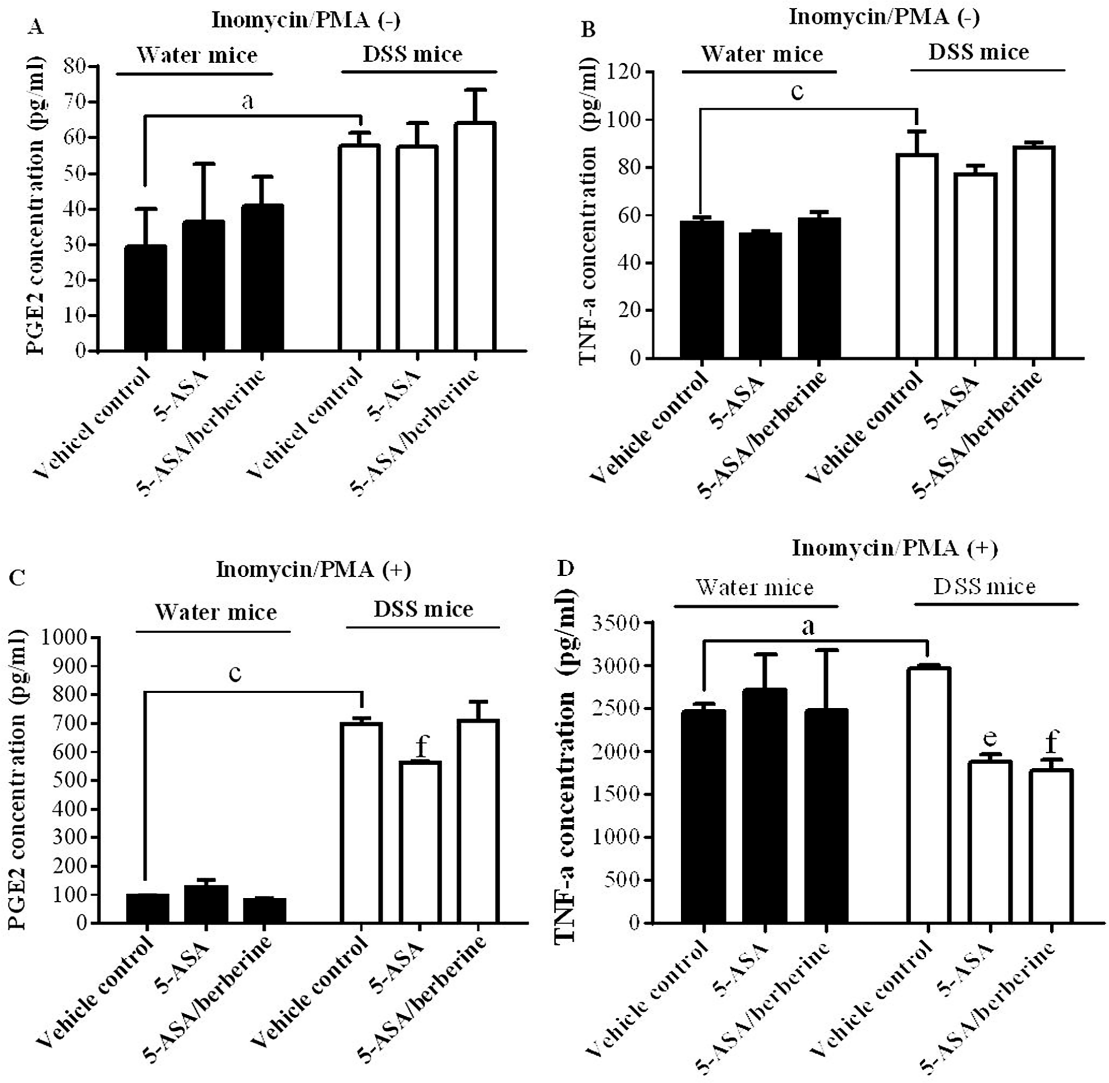
Effects of the addition of berberine to 5-ASA on PGE2 production and TNF-α secretion of lymphocytes. Experimental colitis was induced by giving mice three cycles of DSS. Each cycle consisted of 2% DSS for five days followed by drinking water for 14 days. Control mice received water alone. At the end of treatment, mice were killed and the lymphocyte were isolated from spleens and then cultured for the measurement of PGE2 production and TNF-α secretion without (A and B) or with PMA/ionomycin re-stimulation (C and D). ^a^ *P* < 0.05,^c^ *P* < 0.001 versus water mice; ^e^ *P* < 0.05, ^f^ *P* < 0.01 versus vehicle control (*n* = 3).

The results demonstrated that the lymphocytes without PMA/ionomycin stimulation did not produce significant levels of PGE2 or TNF-α (*n* = 3; Fig 6A and 6B). Even so, when compared with that of water control mice, PGE2 and TNF-α originated from lymphocytes isolated from DSS mice were significantly up-regulated, suggesting an *in vivo* inflammatory response of mice to DSS challenge (Fig 6A and 6B). Either *in vitro* 5-ASA alone or 5-ASA plus berberine treatment did not induce any obvious effects on this elevated PGE2 production or TNF-α secretion (Fig 6A and 6B), indicating these *in vitro* treatments had no reversing effect on existing *in vivo* inflammatory response to DSS.

Conversely, PMA plus ionomycin stimulated lymphocytes of DSS-treated mice showed a significantly higher PGE2 production as compared to that of the water-treated animals (*n* = 3; Fig 6C). 5-ASA alone but not 5-ASA plus berberine treatment significantly reduced PGE2 production of lymphocyte from the DSS mice group (*n* = 3; Fig 6C), indicating berberine did not show additive effects, but might antagonize the action of 5-ASA on inhibiting lymphocyte PGE2 production. Differently, PMA/ionomycin induced similar degree of TNF-α secretion from lymphocytes isolated from both water and DSS-treated group, even the latter showed a significantly higher level as compared to the water-treated animals (*n* = 3; Fig 6D). Interestingly, both two *in vitro* treatments reversed the up-regulation of TNF-α with the combo group more significantly in the DSS but not in water mice when compared to vehicle control (*n* = 3; Fig 6D), suggesting a more potent effect was induced by the addition of berberine to 5-ASA. These dual directions of combination effect on PGE2 and TNF-α may partial explain why only marginal better effect on improvement of disease severity of colitis was induced by 5-ASA plus berberine.

### Drug accumulation in serum, colon and spleen and potential toxicity to major organs after 5-ASA/berberine chronic administration

**Fig 7:**
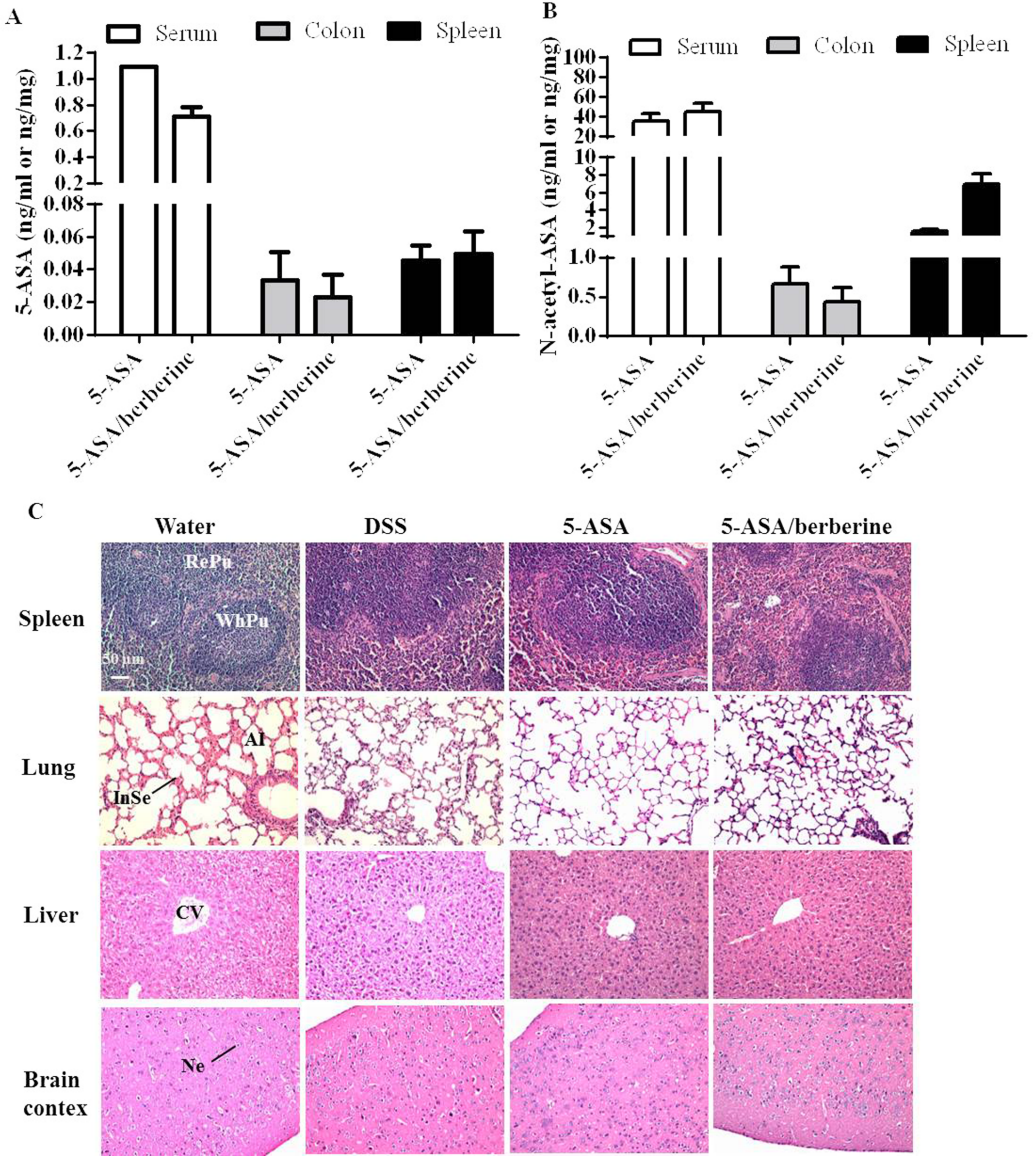
Potential drug accumulation and toxicity of addition of the berberine to 5-ASA in DSS-induced chronic relapsing colitis mice. Experimental colitis was induced by giving mice three cycles of DSS. Each cycle consisted of 2% DSS for five days followed by drinking water for 14 days. The first and last day of DSS treatment were designated as days 0 and 43, respectively. From day 13, experimental mice were treated daily with 5-ASA (200 mg/kg) alone or 5-ASA plus berberine hydrochloride (20 mg/kg) through gavage of 0.2 ml of the respective solution during the 30 days of concomitant DSS-colitis induction. Control mice received water alone. At the end of treatment, mice were killed and the concentrations of 5-ASA (A) and Ac-5-ASA (B) in serum, colon and spleen of colitis mice were measured by LC-MS analysis. Paraffin sections of spleen, lung, liver, and brain (C) were stained with hematoxylin and eosin (H&E). *n* = 4 mice per treatment group. Scale bar = 50 μm. RePu: Red pulp. WhPu: white pulp. Al: alveolus. InSe: interalveolar septum.CV: central vein. Ne: neuron.

Concentrations of 5-ASA and its metabolite Ac-5-ASA in the body are relatively constant along a chronic administration [26,27]. Thus effect of berberine addition to 5-ASA on the drug accumulation were evaluated at the end of study by measuring drug concentration at this time point. As shown in Fig 7A, the presence of berberine did not significantly change the concentration of 5-ASA in serum, colon or spleen of colitis mice. Similarly, the drug concentration of Ac-5-ASA in serum, colon or spleen were not significantly affected by con-current administration of berberine in contrast to the oral administration of 5-ASA alone (*n* = 4; Fig 7B).

The potential toxicity of berberine addition to 5-ASA after chronic administration in DSS mice was evaluated in parallel. There were no apparent clinical signs of toxicity in any treatment group (data not shown). Upon microscopic examination of selected organs of mice, the primary histopathological finding during the 43-day of the study was the presence, in the DSS challenged groups, mononuclear cell infiltrates in the spleen and the resultant enlargement (*n* = 4; Fig 7C). However, no clearly treatment-related findings were observed in either 5-ASA alone or 5-ASA plus berberine treatment groups (Fig 7C). Histopathological examinations of the remaining organs of DSS mice revealed that there were no patterns of clinically important abnormalities in the liver, lung and brain detected (Fig 7C). Accordingly, in mice treated for 30 days with 5-ASA alone or 5-ASA plus berberine, no gross abnormality was seen in the morphologies features consistencies and appearances of above organs (Fig 7C).

## Discussion

Berberine has been used in the treatment of various infectious disorders in CHM for more than 3000 years. It has been recently proven to regulate the activity of Th1 and Th17 cells that play crucial roles in pro-inflammatory response and therefore have been studied as therapeutic strategy in auto-immune diseases including UC [14,28,29]. Our recent study also demonstrated that berberine significantly inhibited Th17 responses in the DSS-induced chronic colitis model in mice (data not published). We therefore speculated that an addition of berberine to 5-ASA could be more effective than 5-ASA alone to reduce intestinal inflammation in a chronic relapsing DSS model mimicking UC of humans.

In our experiments, DSS-induced chronic relapsing colitis was clinically characterized by fluctuating appearance of diarrhea and bloody feces, as well as significant decreases in body weight and colon length in C57BL/6 mice. Notably, in our study 30-day treatment with a sub-clinical dose of 5-ASA plus berberine significantly and stably reduced disease severity of colitis as demonstrated by DAI and histological examinations. It is worthy of note that the clinically effective dose of 5-ASA (4.8 g/d for patients, corresponding to 800 mg/kg/d for mice) is much higher than those used in our present study (200 mg/kg/d) [6]. The addition of berberine to such low level of 5-ASA caused a comparable alleviation of DSS-induced colitis resulting from other alternative therapies such as probiotics [30,31], indicating the effect of 5-ASA is potentiated by the addition of berberine.

To characterize how 5-ASA efficacy was potentiated, their effects on pro-inflammatory responses of colon were monitored. As previously reported, berberine had anti-inflammatory properties in UC experimental models by reducing colonic expression of COX-2 and TNF-α [13]. Meanwhile, 5-ASA proves to be a weak, non-selective inhibitor of COX-2 [32]. In this way, berberine might be able to attenuate the increase of COX-2 expression and show additive effect on reducing other inflammatory cytokines, thereby potentiating the protective effect of 5-ASA against UC. In this work, we showed that the induction of colitis by DSS administration caused a clear pro-inflammatory response (increase of COX-2, TNF-α, IL-12b, and IL-23 mRNAs) in the colon, which was significantly down-regulated after the administration of 5-ASA plus berberine.

NFκB and JAK/STAT signaling are two key pathways which contain transcription factors involved in pro-inflammatory responses in the intestinal inflammation of IBD [12,33]. Therapeutic intervention against NFκB and JAK activation has been reported as a useful strategy for treatment of IBD [12,34–39]. In fact, inhibition of NFκB activity has been suggested to be a major component of the anti-inflammatory activity of 5-ASA [40–43]. Consistently, we found that 5-ASA alone inhibited NFκB but not IKK activation. Further, berberine addition to 5-ASA showed an additive effect on NFκB activation, which might be correlated with an additive inhibitory effect of the co-treatment on the NFκB pathway generated also by berberine [44–46].

Differently, JAK2 phosphorylation was reduced by the combined treatment but not by 5-ASA alone, suggesting that inhibition of the JAK/STAT pathway is likely to participate in the effect of 5-ASA plus berberine against chronic colitis. Previous studies suggest that the JAK/STAT signaling could be an effective therapeutic target for drug intervention due to its apparently unrestrained activation in UC [12,37]. In addition to the regulation of STAT3 [47], berberine may exert an inhibitory effect along the entire JAK/STAT signaling cascade and thus addictive benefits provided by the combined therapy should be considered. Taking together, we observed that dual therapy of berberine plus 5-ASA was more efficient to reduce colon NFκB and JAK activations than 5-ASA alone. Use of berberine as an adjunct therapy may allow the reduction in dose of the mainstream therapy 5-ASA. Furthermore, the direct effect of the combo on pro-inflammatory responses was investigated by their inhibition of *ex-vivo* PGE2 production and TNF-α secretion. Interestingly, additive effect of the combination of 5-ASA with berberine has been observed on TNF-α secretion, but not on PGE2 production, which indicate that the effect of 5-ASA might be potentiated by berberine mainly through their crosstalk on TNF-α mediated inflammatory responses.

It has been reported that 5-ASA undergoes extensive inactivation to N-acetyl-ASA via N-acetyltransferase-1 (NAT-1) in the colonic mucosa and liver, which may be the underlying mechanism leading to low drug availability (20–30%) [48,49]. Studies also showed that berberine markedly decreases the activity of NAT-1 in human colon and bladder tumor cells [50,51]. Therefore, there is a possibility that by inhibiting the activity of NAT-1, berberine can decrease the level of 5-ASA transformation thereby increase level of 5-ASA available to colon, which may contribute to the increased effect of 5-ASA. However, in the present study, we observed that although berberine altered 5-ASA therapy, but did not induce obvious drug accumulation. After a 30-day of drug administration, comparable residual drug concentrations (both 5-ASA and its metabolite Ac-5-ASA) but more potent treatment effects were observed in groups receiving the subclinical dose of 5-ASA in combination with berberine than 5-ASA alone. These data suggest that the co-treatment with berberine did not significantly affect the washout kinetics of 5-ASA. However, the off-drug plasma and tissues sampling was performed at only one time point, additional studies with more frequent plasma sampling for a systematic pharmacokinetics study will further refine the estimates of the influence of berberine on the elimination of 5-ASA and thus its contribution to the more potent treatment effects.

In addition, berberine addition to the subclinical dose (200 mg/kg/day) of 5-ASA which produces pharmacological activity in the chronic colitis model appears to be well tolerated. DSS at the percentage of 2% administered chronically produced enlargements of spleen accompanied by inflammatory changes. It should be noted that the inflammatory changes of spleen observed in DSS mice appear to not be aggravated by this combination of 5-ASA and berberine. In addition, no toxicity was detected on three other selected organs (lung, liver and brain) by all treatments. As berberine is also an “over the counter” therapy, it is an important finding to know that berberine addition to 5-ASA did not worsen disease. Notably, the chronic administration of berberine to patients has been found safe and show beneficial effect on human bodies [52,53]. Actually, berberine exhibits a median lethal dose (LD_50_) value (29586 mg/kg) approximately 100 times higher than that of 5-ASA (3370 mg/kg) when it is orally administered to mice [54]. Thus with comparable anti-UC activity but much lower toxicity, berberine may act as a good adjuvant alternative to 5-ASA in the treatment of UC for reducing toxicity of a chronic 5-ASA administration. A better understanding of the potential toxicity (such as the LD_50_ value and sub-acute toxicity) behind the effects of berberine addition to 5-ASA on UC is warranted to provide a more scientific basis to offer advice to UC patients.

## Conclusion

In summary, both 5-ASA, the mainstay of therapy for UC, and alternative medicine berberine have been proven to be effective in the mouse model of UC. However, it is hard to predict how the combined therapy might interact in a complex manner by affecting each other’s efficacy, bioavailability, metabolism and toxicity. Thus, in this regard, our present study shows no clear adverse interaction between these two drugs. Although there is no obvious adverse interaction, it should be noted that the effect of the combined therapy is modest, which is a marginally better than 5-ASA alone. Therefore, the results from our study suggest that the addition of berberine to 5-ASA have a place in the therapy of UC and provide the rationale for advancement of this combination into clinical development.

## Abbreviations

5-ASA: 5-aminosalicylic acid
CD: Crohn’s disease
CHM: Chinese herbal medicines
DAI: disease activity index
DSS: dextran sodium sulfate
ELISA: enzyme-linked immunosorbent assay
Gapdh: glyceraldehyde 3-phosphate dehydrogenase
H&E: hematoxylin & eosin
IBD: inflammatory bowel disease
NAT-1: N-acetyltransferase-1
PGE2: prostaglandin E2
PMA: phorbol 12-myristate 13-acetate
RT-qPCR: quantitative real-time reverse-transcription polymerase chain reaction
UC: ulcerative colitis
UHPLC-QQQ-MS: ultra-high-performance liquid chromatography

## References

[1] Mehta SJ, Silver AR, Lindsay JO (2013) Review article: strategies for the management of chronic unremitting ulcerative colitis. Alimentary Pharmacology & Therapeutics 38: 77–97. 23718288 10.1111/apt.12345

[2] Cottone M, Renna S, Modesto I, Orlando A (2011) Is 5-ASA still the treatment of choice for ulcerative colitis? Current Drug Targets 12: 1396–1405. 21466493 10.2174/138945011796818126

[3] Zhao LN, Li JY, Yu T, Chen GC, Yuan YH, Chen QK (2014) 5-Aminosalicylates reduce the risk of colorectal neoplasia in patients with ulcerative colitis: an updated meta-analysis. PLoS One 9: e94208 10.1371/journal.pone.0094208 24710620 PMC3978022

[4] Bantel H, Berg C, Vieth M, Stolte M, Kruis W, Schulze-Osthoff K (2000) Mesalazine inhibits activation of transcription factor NF-kappaB in inflamed mucosa of patients with ulcerative colitis. The American Journal of Gastroenterology 95: 3452–3457. 11151876 10.1111/j.1572-0241.2000.03360.x

[5] Sharon P, Ligumsky M, Rachmilewitz D, Zor U (1978) Role of prostaglandins in ulcerative colitis. Enhanced production during active disease and inhibition by sulfasalazine. Gastroenterology 75: 638–640. 30669

[6] Katz S, Lichtenstein GR, Safdi MA (2010) 5-ASA dose-response: maximizing efficacy and adherence. Gastroenterology & Hepatology 6: 1–16. PMC2886460 20567558

[7] Zhang C, Jiang M, Lu A (2013) Considerations of traditional Chinese medicine as adjunct therapy in the management of ulcerative colitis. Clinical Reviews in Allergy & Immunology 44: 274–283. 22669756 10.1007/s12016-012-8328-9

[8] Tillhon M, Guaman Ortiz LM, Lombardi P, Scovassi AI (2012) Berberine: new perspectives for old remedies. Biochemical Pharmacology 84: 1260–1267. 10.1016/j.bcp.2012.07.018 22842630

[9] Derosa G, Maffioli P (2014) Alkaloids in the nature: pharmacological applications in clinical practice of berberine and mate tea. Current Topics in Medicinal Chemistry 14: 200–206. 24359201 10.2174/1568026613666131213155252

[10] Chen CQ, Yu Z, Li YY, Fichna J, Storr M (2014) Effects of berberine in the gastrointestinal tract—a review of actions and therapeutic implications. American Journal of Chinese Medicine 42: 1053–1070. 10.1142/S0192415X14500669 25183302

[11] Qin X, Guo BT, Wan B, Fang L, Lu L, Wu L, et al (2010) Regulation of Th1 and Th17 cell differentiation and amelioration of experimental autoimmune encephalomyelitis by natural product compound berberine. Journal of Immunology 185: 1855–1863. 10.4049/jimmunol.0903853 20622114

[12] Coskun M, Salem M, Pedersen J, Nielsen OH (2013) Involvement of JAK/STAT signaling in the pathogenesis of inflammatory bowel disease. Pharmacological Research 76: 1–8. 10.1016/j.phrs.2013.06.007 23827161

[13] Lee IA, Hyun YJ, Kim DH (2010) Berberine ameliorates TNBS-induced colitis by inhibiting lipid peroxidation, enterobacterial growth and NF-kappaB activation. European Journal of Pharmacology 648: 162–170. 10.1016/j.ejphar.2010.08.046 20828550

[14] Hong T, Yang Z, Lv CF, Zhang Y (2012) Suppressive effect of berberine on experimental dextran sulfate sodium-induced colitis. Immunopharmacology and Immunotoxicology 34: 391–397. 10.3109/08923973.2011.609887 22564173

[15] Yan F, Wang L, Shi Y, Cao H, Liu L, Washington MK, et al (2012) Berberine promotes recovery of colitis and inhibits inflammatory responses in colonic macrophages and epithelial cells in DSS-treated mice. American Journal of Physiology Gastrointestinal and Liver Physiology 302: G504–514. 10.1152/ajpgi.00312.2011 22173918 PMC3311435

[16] Zhou H, Mineshita S (2000) The effect of berberine chloride on experimental colitis in rats in vivo and in vitro. Journal of Pharmacology and Experimental Therapeutics 294: 822–829. 10945829

[17] ClinicalTrials.gov (2015) Berberine chloride in treating patients with ulcerative colitis in remission. [Online] Available from https://clinicaltrialsgov/ct2/show/NCT02365480/) NCT02365480.

[18] Wirtz S, Neufert C, Weigmann B, Neurath MF (2007) Chemically induced mouse models of intestinal inflammation. Nature Protocols 2: 541–546. 17406617 10.1038/nprot.2007.41

[19] Viennois E, Chen F, Laroui H, Baker MT, Merlin D (2013) Dextran sodium sulfate inhibits the activities of both polymerase and reverse transcriptase: lithium chloride purification, a rapid and efficient technique to purify RNA. BMC Research Notes 6: 360 10.1186/1756-0500-6-360 24010775 PMC3847706

[20] Pastorini E, Locatelli M, Simoni P, Roda G, Roda E, Roda A (2008) Development and validation of a HPLC-ESI-MS/MS method for the determination of 5-aminosalicylic acid and its major metabolite N-acetyl-5-aminosalicylic acid in human plasma. Journal of Chromatography B 872: 99–106. 10.1016/j.jchromb.2008.07.026 18691952

[21] Sayer B, Lu J, Green C, Soderholm JD, Akhtar M, McKay DM (2002) Dextran sodium sulphate-induced colitis perturbs muscarinic cholinergic control of colonic epithelial ion transport. British Journal of Pharmacology 135: 1794–1800. 11934821 10.1038/sj.bjp.0704633 PMC1573298

[22] Einerhand AW, Renes IB, Makkink MK, van der Sluis M, Buller HA, Dekker J (2002) Role of mucins in inflammatory bowel disease: important lessons from experimental models. European Journal of Gastroenterology & Hepatology 14: 757–765. 12169985 10.1097/00042737-200207000-00008

[23] Shirazi T, Longman RJ, Corfield AP, Probert CS (2000) Mucins and inflammatory bowel disease. Postgraduate Medical Journal 76: 473–478. 10908374 10.1136/pmj.76.898.473 PMC1741691

[24] Rhodes JM (1997) Mucins and inflammatory bowel disease. QJM 90: 79–82. 9068798 10.1093/qjmed/90.2.79

[25] Yan F, Polk DB (1999) Aminosalicylic acid inhibits IkappaB kinase alpha phosphorylation of IkappaBalpha in mouse intestinal epithelial cells. The Journal of Biological Chemistry 274: 36631–36636. 10593965 10.1074/jbc.274.51.36631

[26] Staerk Laursen L, Stokholm M, Bukhave K, Rask-Madsen J, Lauritsen K (1990) Disposition of 5-aminosalicylic acid by olsalazine and three mesalazine preparations in patients with ulcerative colitis: comparison of intraluminal colonic concentrations, serum values, and urinary excretion. Gut 31: 1271–1276. 2253912 10.1136/gut.31.11.1271 PMC1378698

[27] Tzivras M, Konstandinidis A, Hatzis G, Paraskeva K, Skandalis N, Archimandritis A (1997) Systemic absorption of 5-aminosalicylic acid in patients with inactive ulcerative colitis treated with olsalazine and mesalazine. European Journal of Gastroenterology & Hepatology 9: 729–730. 9262986 10.1097/00042737-199707000-00016

[28] Kuo CL, Chi CW, Liu TY (2004) The anti-inflammatory potential of berberine in vitro and in vivo. Cancer Letters 203: 127–137. 14732220 10.1016/j.canlet.2003.09.002

[29] Cui G, Qin X, Zhang Y, Gong Z, Ge B, Zang YQ (2009) Berberine differentially modulates the activities of ERK, p38 MAPK, and JNK to suppress Th17 and Th1 T cell differentiation in type 1 diabetic mice. The Journal of Biological Chemistry 284: 28420–28429. 10.1074/jbc.M109.012674 19661066 PMC2788891

[30] Toumi R, Soufli I, Rafa H, Belkhelfa M, Biad A, Touil-Boukoffa C (2014) Probiotic bacteria lactobacillus and bifidobacterium attenuate inflammation in dextran sulfate sodium-induced experimental colitis in mice. International Journal of Immunopathology and Pharmacology 27: 615–627. 25572742 10.1177/039463201402700418

[31] Toumi R, Abdelouhab K, Rafa H, Soufli I, Raissi-Kerboua D, Djeraba Z, et al (2013) Beneficial role of the probiotic mixture Ultrabiotique on maintaining the integrity of intestinal mucosal barrier in DSS-induced experimental colitis. Immunopharmacology and Immunotoxicology 35: 403–409. 10.3109/08923973.2013.790413 23638770

[32] Warner TD, Giuliano F, Vojnovic I, Bukasa A, Mitchell JA, Vane JR (1999) Nonsteroid drug selectivities for cyclo-oxygenase-1 rather than cyclo-oxygenase-2 are associated with human gastrointestinal toxicity: A full in vitro analysis. Proceedings of the National Academy of Sciences of the United States of America 96: 7563–7568. 10377455 10.1073/pnas.96.13.7563 PMC22126

[33] Andresen L, Jorgensen VL, Perner A, Hansen A, Eugen-Olsen J, Rask-Madsen J (2005) Activation of nuclear factor kappaB in colonic mucosa from patients with collagenous and ulcerative colitis. Gut 54: 503–509. 15753535 10.1136/gut.2003.034165 PMC1774469

[34] Ardite E, Panes J, Miranda M, Salas A, Elizalde JI, Sans M, et al (1998) Effects of steroid treatment on activation of nuclear factor kappa B in patients with inflammatory bowel disease. British Journal of Pharmacology 124: 431–433. 9647464 10.1038/sj.bjp.0701887 PMC1565427

[35] Shah YM, Ma X, Morimura K, Kim I, Gonzalez FJ (2007) Pregnane X receptor activation ameliorates DSS-induced inflammatory bowel disease via inhibition of NF-kappaB target gene expression. American Journal of Physiology Gastrointestinal and Liver Physiology 292: G1114–1122. 17170021 10.1152/ajpgi.00528.2006

[36] Li J, Zhong W, Wang W, Hu S, Yuan J, Zhang B, et al (2014) Ginsenoside metabolite compound K promotes recovery of dextran sulfate sodium-induced colitis and inhibits inflammatory responses by suppressing NF-kappaB activation. PLoS One 9: e87810 10.1371/journal.pone.0087810 24504372 PMC3913696

[37] Tao F, Qian C, Guo W, Luo Q, Xu Q, Sun Y (2013) Inhibition of Th1/Th17 responses via suppression of STAT1 and STAT3 activation contributes to the amelioration of murine experimental colitis by a natural flavonoid glucoside icariin. Biochemical Pharmacology 85: 798–807. 10.1016/j.bcp.2012.12.002 23261528

[38] Panes J, Su C, Bushmakin AG, Cappelleri JC, Mamolo C, Healey P (2015) Randomized trial of tofacitinib in active ulcerative colitis: analysis of efficacy based on patient-reported outcomes. BMC Gastroenterology 15: 14 10.1186/s12876-015-0239-9 25651782 PMC4323227

[39] Liu L, Liu YL, Liu GX, Chen X, Yang K, Yang YX, et al (2013) Curcumin ameliorates dextran sulfate sodium-induced experimental colitis by blocking STAT3 signaling pathway. International Immunopharmacology 17: 314–320. 10.1016/j.intimp.2013.06.020 23856612

[40] Wahl C, Liptay S, Adler G, Schmid RM (1998) Sulfasalazine: a potent and specific inhibitor of nuclear factor kappa B. The Journal of Clinical Investigation 101: 1163–1174. 9486988 10.1172/JCI992 PMC508669

[41] Kaiser GC, Yan F, Polk DB (1999) Mesalamine blocks tumor necrosis factor growth inhibition and nuclear factor kappaB activation in mouse colonocytes. Gastroenterology 116: 602–609. 10029619 10.1016/s0016-5085(99)70182-4 PMC3606885

[42] Liptay S, Bachem M, Hacker G, Adler G, Debatin KM, Schmid RM (1999) Inhibition of nuclear factor kappa B and induction of apoptosis in T-lymphocytes by sulfasalazine. British Journal of Pharmacology 128: 1361–1369. 10602313 10.1038/sj.bjp.0702937 PMC1571782

[43] Mbodji K, Charpentier C, Guerin C, Querec C, Bole-Feysot C, Aziz M, et al (2013) Adjunct therapy of n-3 fatty acids to 5-ASA ameliorates inflammatory score and decreases NF-kappaB in rats with TNBS-induced colitis. The Journal of Nutritional Biochemistry 24: 700–705. 10.1016/j.jnutbio.2012.03.022 22841543

[44] Fu K, Lv X, Li W, Wang Y, Li H, Tian W, et al (2015) Berberine hydrochloride attenuates lipopolysaccharide-induced endometritis in mice by suppressing activation of NF-kappaB signal pathway. International Immunopharmacology 24: 128–132. 10.1016/j.intimp.2014.11.002 25479718

[45] Gao MY, Chen L, Yang L, Yu X, Kou JP, Yu BY (2014) Berberine inhibits LPS-induced TF procoagulant activity and expression through NF-kappaB/p65, Akt and MAPK pathway in THP-1 cells. Pharmacological Reports 66: 480–484. 10.1016/j.pharep.2013.12.004 24905527

[46] Wang Y (2013) Attenuation of berberine on lipopolysaccharide-induced inflammatory and apoptosis responses in beta-cells via TLR4-independent JNK/NF-kappaB pathway. Pharmaceutical Biology: [Epub ahead of print]. 10.3109/13880209.2013.840851 24188583

[47] Tsang CM, Cheung YC, Lui VW, Yip YL, Zhang G, Lin VW, et al (2013) Berberine suppresses tumorigenicity and growth of nasopharyngeal carcinoma cells by inhibiting STAT3 activation induced by tumor associated fibroblasts. BMC Cancer 13: 619 10.1186/1471-2407-13-619 24380387 PMC3890551

[48] Sandborn WJ, Hanauer SB, Buch A (2004) Comparative pharmacokinetics of equimolar doses of 5-aminosalicylate administered as oral mesalamine (Asacol) and balsalazide: a randomized, single-dose, crossover study in healthy volunteers. Alimentary Pharmacology & Therapeutics 19: 1089–1098. 15142198 10.1111/j.1365-2036.2004.01964.x

[49] Gu GZ, Xia HM, Pang ZQ, Liu ZY, Jiang XG, Chen J (2011) Determination of sulphasalazine and its main metabolite sulphapyridine and 5-aminosalicylic acid in human plasma by liquid chromatography/tandem mass spectrometry and its application to a pharmacokinetic study. Journal of Chromatography B 879: 449–456. 10.1016/j.jchromb.2010.12.034 21251889

[50] Lin JG, Chung JG, Wu LT, Chen GW, Chang HL, Wang TF (1999) Effects of berberine on arylamine N-acetyltransferase activity in human colon tumor cells. The American Journal of Chinese Medicine 27: 265–275. 10467460 10.1142/S0192415X99000306

[51] Chung JG, Wu LT, Chu CB, Jan JY, Ho CC, Tsou MF, et al (1999) Effects of berberine on arylamine N-acetyltransferase activity in human bladder tumour cells. Food and Chemical Toxicology 37: 319–326. 10418949 10.1016/s0278-6915(99)00016-2

[52] Lan J, Zhao Y, Dong F, Yan Z, Zheng W, Fan J, et al (2015) Meta-analysis of the effect and safety of berberine in the treatment of type 2 diabetes mellitus, hyperlipemia and hypertension. Journal of Ethnopharmacology 161: 69–81. 10.1016/j.jep.2014.09.049 25498346

[53] Yin J, Xing H, Ye J (2008) Efficacy of berberine in patients with type 2 diabetes mellitus. Metabolism: Clinical and Experimental 57: 712–717. 18442638 10.1016/j.metabol.2008.01.013 PMC2410097

[54] Kheir MM, Wang Y, Hua L, Hu J, Li L, Lei F, et al (2010) Acute toxicity of berberine and its correlation with the blood concentration in mice. Food and Chemical Toxicology 48: 1105–1110. 10.1016/j.fct.2010.01.033 20138204

